# Dietary magnesium supplementation in cats with chronic kidney disease: A prospective double‐blind randomized controlled trial

**DOI:** 10.1111/jvim.17134

**Published:** 2024-07-01

**Authors:** Pak‐Kan Tang, Dirk Hendrik Nicolaas van den Broek, Rosanne E. Jepson, Rebecca F. Geddes, Yu‐Mei Chang, Nicola Lötter, Delphine Moniot, Vincent Biourge, Jonathan Elliott

**Affiliations:** ^1^ Department of Comparative Biomedical Sciences, Royal Veterinary College University of London London United Kingdom; ^2^ Department of Clinical Sciences, Faculty of Veterinary Medicine Utrecht University Utrecht The Netherlands; ^3^ Department of Clinical Science and Services, Royal Veterinary College University of London London United Kingdom; ^4^ Research Support Office, Royal Veterinary College University of London London United Kingdom; ^5^ Royal Canin Research Center Aimargues France; ^6^ Present address: Department of Comparative Biomedical Sciences Royal Veterinary College, University of London London United Kingdom

**Keywords:** anti‐calcemic, calcium, CKD‐MBD, fibroblast growth factor‐23, hypercalcemia, magnesium oxide

## Abstract

**Background:**

Plasma total magnesium concentration (tMg) is a prognostic indicator in cats with chronic kidney disease (CKD), shorter survival time being associated with hypomagnesemia. Whether this risk factor is modifiable with dietary magnesium supplementation remains unexplored.

**Objectives:**

Evaluate effects of a magnesium‐enriched phosphate‐restricted diet (PRD) on CKD–mineral bone disorder (CKD‐MBD) variables.

**Animals:**

Sixty euthyroid client‐owned cats with azotemic CKD, with 27 and 33 allocated to magnesium‐enriched PRD or control PRD, respectively.

**Methods:**

Prospective double‐blind, parallel‐group randomized trial. Cats with CKD, stabilized on a PRD, without hypermagnesemia (tMg >2.43 mg/dL) or hypercalcemia (plasma ionized calcium concentration, (iCa) >6 mg/dL), were recruited. Both intention‐to‐treat and per‐protocol (eating ≥50% of study diet) analyses were performed; effects of dietary magnesium supplementation on clinicopathological variables were evaluated using linear mixed effects models.

**Results:**

In the per‐protocol analysis, tMg increased in cats consuming a magnesium‐enriched PRD (β, 0.25 ± .07 mg/dL/month; *P* < .001). Five magnesium supplemented cats had tMg >2.92 mg/dL, but none experienced adverse effects. Rate of change in iCa differed between groups (*P* = .01), with decreasing and increasing trends observed in cats fed magnesium‐enriched PRD and control PRD, respectively. Four control cats developed ionized hypercalcemia versus none in the magnesium supplemented group. Log‐transformed plasma fibroblast growth factor‐23 concentration (FGF23) increased significantly in controls (β, 0.14 ± .05 pg/mL/month; *P* = .01), but remained stable in the magnesium supplemented group (β, 0.05±.06 pg/mL/month; *P* =.37).

**Conclusions and Clinical Importance:**

Magnesium‐enriched PRD is a novel therapeutic strategy for managing feline CKD‐MBD in cats, further stabilizing plasma FGF23 and preventing hypercalcemia.

**Abbreviations**
β: coefficient
ALT: alanine aminotransferase
ARB: angiotensin II receptor blocker
BCS: body condition score
Ca : P: calcium‐to‐phosphorus ratio
CKD: chronic kidney disease
CKD‐MBD: chronic kidney disease–mineral and bone disorder
CONSORT: Consolidated Standards of Reporting Trials
EDTA: ethylenediaminetetraacetic acid
FGF23: fibroblast growth factor‐23
HCO_3_^−^
: bicarbonate
iCa: ionized calcium concentration
IRIS: International Renal Interest Society
ln: natural logarithm
ln[FGF23]: log‐transformed fibroblast growth factor‐23
ln[PTH]: log‐transformed parathyroid hormone
MCS: muscle condition score
MgO: magnesium oxide
PRD: phosphate‐restricted diet
PTH: parathyroid hormone
SBP: systolic blood pressure
SDMA: symmetric dimethylarginine
tCa: total calcium concentration
tMg: total magnesium concentration
USG: urine specific gravity
UTI: urinary tract infection
VC: vascular calcification

## 1 INTRODUCTION

Increasing evidence in both the human and veterinary medical literature has suggested that magnesium influences disease progression and mortality among patients with chronic kidney disease (CKD).^1^, ^2^, ^3^, ^4^, ^5^, ^6^ Hypomagnesemia was a common electrolyte abnormality among 5126 human patients with stage 1 to 5 CKD.^7^

Magnesium is a potent calcification inhibitor^8^; hypomagnesemia is associated with vascular calcification (VC) and increased risk of cardiovascular events in rodent models and human CKD patients.^4^, ^9^ Dietary magnesium supplementation prevented VC in rat CKD models,^1^, ^10^ and increased serum magnesium concentration in hemodialysis patients is associated with decreased mortality risk.^11^

Magnesium appears to be involved in calcium and fibroblast growth factor‐23 (FGF23) regulation. Rats fed a magnesium‐deficient diet (0.005% w/w) for 21 days had significantly lower serum magnesium concentration and higher serum calcium and FGF23 concentrations, compared with their counterparts fed a control diet (0.051% w/w).^12^ Furthermore, an inverse relationship between serum magnesium concentration and FGF23 was identified in a cross‐sectional study involving 255 human CKD patients undergoing hemodialysis.^13^ Similar findings between these 2 variables also were observed in cats with azotemic CKD,^5^ and hypomagnesemia was associated with increased risk of death.^5^

Oral administration of magnesium significantly lowers serum FGF23^13^ and calcium concentrations^14^ in human patients receiving hemodialysis. However, the effects of magnesium supplementation on CKD–mineral and bone disorder (CKD‐MBD) in cats have never been evaluated. Because increasing plasma total calcium concentration (tCa) and FGF23 excess are associated with CKD progression in cats,^15^ we hypothesized that dietary magnesium supplementation would improve CKD‐MBD in cats by stabilizing FGF23 and calcium disturbances.

Our prospective randomized controlled trial was designed, first, to determine the effect of dietary magnesium supplementation on plasma total magnesium concentration (tMg) in cats with azotemic CKD; and, second, to evaluate changes in clinicopathological variables associated with CKD‐MBD in relation to dietary magnesium supplementation.

## 2 METHODS

### 2.1 Study design

A prospective, double‐blinded, parallel‐group randomized controlled dietary trial (referred to herein as MAGMA) was designed to evaluate the effects of a magnesium‐enriched phosphate‐restricted diet (PRD), containing additional magnesium oxide (MgO), on CKD‐MBD variables when fed for 12 to 16 weeks to cats with azotemic CKD. The study was conducted at the Royal Veterinary College (Hatfield, UK) held at 2 first‐opinion practices in London from November 1, 2017 to March 28, 2023. Ethical approval for our study protocol was granted by the Royal Veterinary College Ethics and Welfare Committee (URN20171713‐3). A participant information sheet was provided and owner informed consent was obtained before study enrollment (Tables S1 and S2). The study was funded by Royal Canin SAS (Aimargues, France), who manufactured both diets (control and magnesium‐enriched PRD; see Table 1 for diet composition) with the dietary design based on the results obtained from a pilot study.^16^ The funder did not participate in data collection or analyses.

**TABLE 1:** Nutritional composition and ingredients for the diets in this prospective dietary trial (MAGMA).

Nutrient	Control PRD		Magnesium‐enriched PRD	
	Dry diet	Wet diet	Dry diet	Wet diet
Moisture (%)	5.5	77	5.5	77
Protein (g/Mcal)	55.2	62.9	55.4	62.9
Fat content (g/Mcal)	41.08	62.89	41.21	62.89
Crude fiber (g/Mcal)	11.54	3.69	11.58	3.69
Crush ash (g/Mcal)	14.37	10.22	15.20	10.22
Dietary fiber (g/Mcal)	24.21	6.29	24.30	6.29
Calcium (g/Mcal)	1.49	1.10	1.50	1.10
Phosphorus (g/Mcal)	0.77	0.67	0.78	0.67
Ca : P	1.9	1.6	1.9	1.6
Sodium (g/Mcal)	0.98	0.86	0.98	0.86
Magnesium (g/Mcal)	0.18	0.13	0.62	0.62
Potassium (g/Mcal)	2.21	1.81	2.22	1.81
Vitamin D (IU/Mcal)	206.70	353.77	207.50	353.77
Metabolizable energy^a^ (kcal/kg)	3383	1180	3868	1180

Abbreviations: Ca : P, calcium‐to‐phosphorus ratio; PRD, phosphate‐restricted diet.

^a^ Calculated according to the National Research Council (NRC) 2006 equation using crude fiber.

### 2.2 Study participants

All enrolled client‐owned cats included previously had been participating in standardized longitudinal observational studies with ethical approval granted by the Royal Veterinary College Ethics and Welfare Committee (URN20131258E). Cats were eligible for MAGMA enrollment if they were diagnosed with azotemic CKD and had normal calcium concentrations (plasma tCa ≤11.8 mg/dL and blood ionized calcium concentration [iCa] ≤6 mg/dL) and had been stabilized on a PRD (Feline Veterinary Diet Renal [dry and wet], Royal Canin SAS, Aimargues, France [dry] and Masterfoods, Bruck, Austria [wet]) with a phosphorus content of 0.7 to 1.1 g/Mcal and calcium‐to‐phosphorus ratio (Ca : P) of 1.3‐2 (see Table S3 for diet composition). This PRD had to be fed at a minimum of 50% in proportion by volume of the total amount of food fed for a minimum of 4 weeks before enrollment. To be included, a cat initially had to have hypomagnesemia (plasma tMg <1.94 mg/dL), but because of low recruitment rates, from May 2019, this cutoff was increased to include cats with normomagnesemia (tMg <2.44 mg/dL).^5^

Cats were excluded if they had suspected hyperthyroidism, plasma total thyroxine concentration >40 nmol/L, were undergoing medical treatment for hyperthyroidism, or had comorbid disease conditions including diabetes mellitus, bacterial urinary tract infection (UTI), food hypersensitivity to poultry (both trial diets were poultry‐based), a history of struvite crystalluria within the previous 12 months, or were receiving corticosteroids, furosemide, bisphosphonates, calcium‐based phosphate binders, or angiotensin II receptor blockers (ARB). Cats with International Renal Interest Society (IRIS) stage 4 CKD with substantial worsening of azotemia (>25% increase in plasma creatinine concentration) at enrollment were excluded from the trial. Cats receiving amlodipine besylate for systemic hypertension were eligible for enrollment, with no changes made to the treatment regimen (ie, dosage of amlodipine) within 14 days before study enrollment.

### 2.3 Randomization and intervention

Eligible cats were randomly assigned to either a magnesium‐enriched PRD (with a magnesium content of 0.62 g/Mcal; 0.2% elemental magnesium) or a control PRD (with a magnesium content of 0.13‐0.18 g/Mcal; 0.07% elemental magnesium) according to a predetermined randomized sequence generated using an online‐based randomization program (QuickCalcs, GraphPad Software, Boston, MA). Block randomization with a block size of 6 cats was used, with treatment allocation ratio set at 1 to 1. Both study diets were designed and manufactured by Royal Canin SAS (Aimargues, France) with identical packaging but labeled differently for the 2 trial diets with code names “AYC” and “XBZ”. Only a dry formulation of the trial diet was available for the 1st 11 months of the trial (November 2017 to September 2018), thereafter both wet and dry formulations were available for the remainder of the study period.

The screening visit was designated as baseline, and the cats enrolled in the study subsequently were fed their assigned diet, with reexamination and blood and urine sampling performed at 4 to 8 weeks (visit 1) and 12 to 16 weeks (visit 2). Owners were encouraged to feed their cats the highest proportion of trial diet possible, but if necessary, feeding a proportion of the original pretrial PRD or a maintenance diet was allowed. A detailed dietary questionnaire (Figures S1 and S2) was completed at all visits to quantify the proportion of different diets consumed. Owners were advised to observe for possible adverse effects associated with hypermagnesemia, including diarrhea or signs of stranguria, hematuria, or dysuria throughout the study. The trial was terminated if moderate hypermagnesemia (defined as tMg >2.92 mg/dL) was documented.

### 2.4 Sample size calculation

Before trial commencement, a pilot study was conducted to evaluate the change in plasma FGF23 concentration after PO administration of magnesium glycinate. Eight cats with azotemic CKD and hypomagnesemia received magnesium glycinate supplementation at 100 mg/kg (18.2 mg/kg elemental magnesium) once daily for 4 weeks.^16^ Three cats did not tolerate or were not given the magnesium salt and therefore served as the control group for comparison. Based on the paired differences (0.55 ± 0.7) on log‐transformed fibroblast growth factor‐23 (lnFGF23) between CKD cats with and without PO magnesium supplementation, a sample size of 60 cats (30 per group) was required to detect the decrease in lnFGF23, with 85% power and 5% type I error rate, in CKD cats fed a magnesium‐enriched PRD compared with the control group.

### 2.5 Data collection

At all visits, physical examination was performed including measurement of body weight, body condition score (BCS; 9‐point scale), and muscle condition score (MCS; 4‐point scale).^17^ Blood samples were collected by jugular venipuncture into heparinized, ethylenediaminetetraacetic acid (EDTA), and plain tubes, whereas urine was obtained by cystocentesis. Measurements of iCa and bicarbonate (HCO_3_^−^) concentrations, and venous pH were obtained immediately using nonanticoagulated whole blood or using electrolyte‐balanced heparinized whole blood within 5 min of venipuncture,^18^ using a point‐of‐care blood analyzer (*i‐STAT 1*, Abbott Point of Care, Inc, Princeton, New Jersey). All samples collected were stored at 4°C for <6 h before centrifugation and separation. Full biochemical analyses were performed on heparinized plasma by an external laboratory (IDEXX laboratories, Wetherby, UK). In‐house urinalyses, including urine specific gravity (USG) measurement by refractometry, dipstick chemistry, and microscopic urine sediment examination, were performed on the day of collection. Bacterial culture (Royal Veterinary College Diagnostic Laboratory Services, Hatfield, UK) was used to confirm UTI. Systolic blood pressure (SBP) was measured by the Doppler method.^19^

Intact FGF23 was measured on stored EDTA plasma samples using a validated enzyme‐linked immunosorbent assay (FGF23 ELISA Kit, Kainos Laboratories, Tokyo, Japan),^20^ and parathyroid hormone (PTH) was measured using either a validated total intact PTH immunoradiometric assay^21^ (IRA; total intact PTH immunoradiometric assay–coated bead version, 3KG600, Scantibodies, Santee, California) or a validated 2‐site immunoenzymatic assay^22^ (IEA; ST AIA‐PACK Intact PTH, Tosoh Bioscience, Tessenderlo, Belgium) for samples collected before or after January 2021, respectively. All PTH measurements from the same cat were obtained using the same assay. Alteration in PTH assay during this trial was caused by the unforeseen discontinuation of the IRA in 2020. The PTH measurements obtained using the 2 assays were comparable (Figure S3). Parathyroid hormone concentrations of 2.6 and 0.55 pg/mL were assigned to samples measured at <5.2 and < 1.1 pg/mL, the lower limit of detection of the IRA and IEA, respectively.^21^, ^22^

### 2.6 Statistical analyses

Statistical analyses were performed using R software (R 4.1.1 GUI 1.77 High Sierra build, R Foundation for Statistical Computing, Vienna, Austria). Type I error rate was set at 0.05. The normality of continuous variables was assessed by visual inspection of Q‐Q plots and using the Shapiro‐Wilk test. Levene's test was used to determine if the groups had equal variances. Most data were not normally distributed and therefore numerical data are presented as median (25th, 75th percentile) for consistency. Categorical data are presented as percentages.

Plasma FGF23 and PTH were log‐transformed (natural logarithm [ln]) for normalization. Independent samples *t‐*test or Mann‐Whitney *U* test was used to compare baseline continuous variables between the diet groups. Comparison of the proportions of categorical outcomes was made using Chi‐squared or Fisher's exact test.

Pearson's or Spearman's correlation was used to evaluate the relationships between baseline tMg and other CKD‐MBD variables, namely tCa, iCa, creatinine, symmetric dimethylarginine (SDMA), urea, phosphate, FGF23, and PTH.

Data were analyzed using both intention‐to‐treat (all cats that were enrolled and randomized into this trial with at least 1 follow‐up visit available) and per‐protocol (only cats eating ≥50% of study diet at revisits were included). The proportion of cats with “uptrend” total and ionized calcium concentrations (defined as tCa and iCa regression gradient >0 by linear regression) between the diet groups was compared using Chi‐squared or Fisher's exact test.

The effects of the study diets on longitudinal biochemical data and SBP measurements from baseline and all follow‐up visits (approximately 4 months) were assessed using linear mixed effects models (R packages <lme4> and < ImerTest>); generalized estimating equations with binomial logistic link function and exchangeable correlation structure (R package <geepack>) and generalized linear mixed model with cumulative logistic link function (R package <ordinal>) were used to assess the change in binary and ordinal variables (ie, BCS and MCS) over time. Body condition score was categorized into 2 levels (“1‐3”, “4‐9”) before analysis because most measurements (96%) were below 6. Group (“magnesium‐enriched PRD” vs “control PRD”), time (in months [30.4 days]) and the interaction between group and time were treated as fixed effects; cat identification and time nested within individual cats were included as 2 uncorrelated random effects. Residuals were assumed to be independent in the model, and normality of the residuals from linear mixed effects models was evaluated by visual inspection of histograms. For BCS, MCS, log‐transformed parathyroid hormone (ln[PTH]), alanine aminotransferase activity (ALT) and venous HCO_3_^−^ in intention‐to‐treat analysis and BCS, MCS, body weight, plasma sodium concentration, ALT and venous pH in per‐protocol analysis, only the case number of each individual cat was included as random effect because of a model convergence issue. Imputation of missing data was not attempted. Results are reported as rate of change coefficient (β) ± SE.

## 3 RESULTS

**FIGURE 1:**
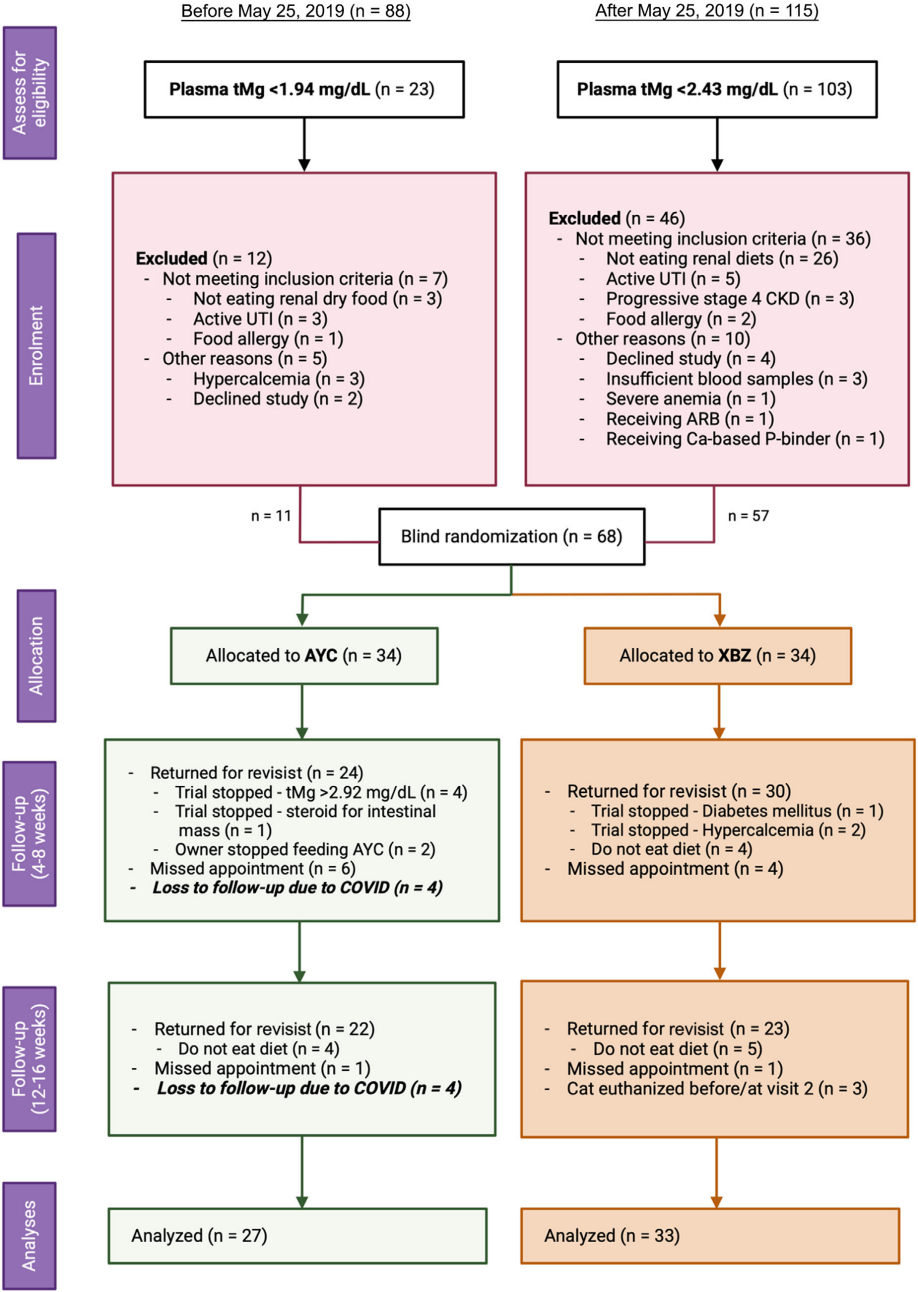
A Consolidated Standards of Reporting Trials (CONSORT) flow diagram of this prospective dietary trial (MAGMA). UTI, urinary tract infection.

A Consolidated Standards of Reporting Trials (CONSORT) flow diagram illustrating progress through different phases of the randomized controlled clinical trial MAGMA is presented in Figure 1. Between November 2017 and May 2019, 88 cats with CKD were screened for study eligibility. Eight (9.1%) and 57 (64.8%) cats were hypermagnesemic and normomagnesemic at screening, respectively, and therefore were not eligible for enrollment at that time. Between June 2019 and December 2022, 115 cats with CKD underwent eligibility screening, including 25 cats that previously had been screened. Twelve cats (10.4%) were hypermagnesemic at screening and therefore not eligible for the trial. Therefore, an additional 57 cats were enrolled, with 29 and 28 cats allocated to a magnesium‐enriched PRD and control PRD, respectively. Details of cats that were excluded from the trial are presented in Figure 1.

Overall, 68 cats, with 34 in each diet group, were recruited into the trial. However, 4 cats (all allocated randomly to the magnesium group with 3 from the same household) recruited in March 2020 had no follow‐up visits because of the COVID‐19 pandemic; another 4 cats were inappropriately enrolled, of which 3 were hypercalcemic at enrollment (Data S1), and 1 cat that normally consumed a diet of exclusively wet food was recruited in December 2017, at a time when only dry formulation of the trial diet was available.

Subsequently, 60 CKD cats met the inclusion and exclusion criteria for the trial, with 27 cats allocated to a magnesium‐enriched PRD (“AYC”) and 33 allocated to a control PRD (“XBZ”). At enrollment, 2 had IRIS stage 1, 46 had IRIS stage 2, 11 had IRIS Stage 3, and 1 had IRIS stage 4 CKD. Domestic shorthair was the most common breed (n = 43), followed by domestic longhair (n = 9) and 1 of each of the following breeds: Bengal, Birman, Burmese, Devon Rex, Norwegian Forest, Siamese, Tiffany, and Tonkinese. Of the 60 cats enrolled, 20 (33.3%) cats were hypomagnesemic (tMg <1.94 mg/dL) at baseline, with 11 and 9 cats allocated to a magnesium‐enriched PRD and control PRD, respectively (*P* = .27).

### 3.1 Comparison of baseline variables between cats in different treatment arms

As shown in Table 2, baseline demographic and clinicopathological characteristics were similar between the 2 study groups, except for plasma ALT activity which was higher in the control group (*P* = .02). However, the proportion of cats with increased ALT activity (defined as >60 U/L) did not differ between the 2 groups (*P* = .16). Thirteen cats were receiving amlodipine besylate to control systemic hypertension at baseline, with no difference in proportion between groups (*P* = .93). No cats received phosphate binders during the trial. For the per‐protocol analysis, 16 cats (26.7%) did not consume a minimum of 50% study diet and therefore were excluded; 44 cats remained for analysis, with 21 and 23 cats allocated to a magnesium‐enriched PRD and a control PRD, respectively, and no difference in any baseline clinicopathological variables between the 2 diet groups was found (Table 3).

**TABLE 2:** Descriptive statistics at enrolment for all randomized cats (n = 60) in this prospective dietary trial (MAGMA), grouped based on the allocated study diet (control PRD vs magnesium‐enriched PRD).

Variables (reference interval)	Control PRD (n = 33)		Magnesium‐enriched PRD (n = 27)		*P*‐value
	Median [25th, 75th Percentile]	n	Median [25th, 75th Percentile]	n	
Age (years)	16.2 [13.3, 17.7]	33	15.2 [13.0, 17.0]	27	.87
BCS (“1‐3”, “4‐9”, n [%])	12 [36], 21 [64]	33	6 [22], 19 [76]	25	.31
MCS (“0”, “1”, “2”, “3”, n [%])	1 [3], 15 [45], 16 [48], 1 [3]	33	1 [4], 7 [28], 12 [48], 5[20]	25	.13
Weight (kg)	3.7 [3.2, 4.3]	33	4.2 [3.3, 4.7]	26	.52
Sex (female neutered, n [%])	17 [52]	33	10 [37]	27	.26
Albumin (2.5‐4.5 g/dL)	3.0 [2.8, 3.2]	33	3.1 [2.9, 3.2]	27	.77
ALP (≤ 60 U/L)	24 [20, 33]	33	26 [20, 33]	27	.88
ALT (5‐60 U/L)	54 [47, 65]	33	43 [34, 54]	27	**.02**
ALT (>60 U/L), n [%]	13 [39]	33	6 [22]	27	.16
Chloride (100‐124 mEq/L)	117 [114, 118]	20	117 [116, 119]	15	.5
Creatinine (0.23‐2 mg/dL)	2.41 [2.12, 2.74]	33	2.39 [2.1, 2.76]	27	.96
FGF23^a^ (56‐700 pg/mL)	418 [212, 801]	32	421 [225, 673]	26	.85
Glucose (54‐117 mg/dL)	114 [101, 128]	30	115 [101, 137]	25	.75
Venous HCO_3_^−^ (17‐24 mEq/L)	22.1 [19.4, 23.6]	28	21.6 [20, 23.3]	24	.41
Hypertension (controlled) (n [%])	7 [21]	33	6 [22]	27	.93
Ionized calcium (4.76‐5.48 mg/dL)	5.32 [5.2, 5.48]	28	5.28 [5.2, 5.4]	24	.36
PCV (30%‐45%)	34 [30, 37]	33	34 [30, 37]	27	.7
Venous pH (7.21‐7.44)	7.37 [7.34, 7.39]	28	7.38 [7.36, 7.39]	24	.23
Phosphate (2.79‐6.81 mg/dL)	3.99 [3.25, 4.4]	33	3.62 [3.25, 3.87]	27	.12
Potassium (3.5‐5.5 mEq/L)	4.03 [3.81, 4.20]	20	4.01 [3.70, 4.22]	25	.64
PTH^a^, ^b^ (2.6‐17.6 pg/mL)	10.3 [7.2, 16.4]	22	11.3 [6.6, 19.8]	20	.69
SBP (<160 mmHg)	132 [121, 143]	33	135 [121, 145]	27	.68
SDMA (1‐14 μg/dL)	18 [16, 21]	33	18 [15, 20]	27	.73
Sodium (145‐157 mEq/L)	154 [152, 155]	20	153 [152, 155]	15	.56
Total calcium (8.2‐11.8 mg/dL)	10.24 [9.84, 10.84]	33	10.12 [9.88, 10.64]	27	.31
Total magnesium (1.73‐2.57 mg/dL)	2.09 [1.87, 2.24]	33	2.04 [1.87, 2.19]	27	.51
Total protein (6.0‐8.0 g/dL)	8.0 [7.5, 8.4]	33	7.5 [7.3, 8.2]	27	.23
Urea (7.0‐27.7 mg/dL)	47.3 [42.6, 52.9]	20	44.8 [39.8, 56.0]	15	1
USG (≥1.035)	1.017 [1.015, 1.018]	17	1.015 [1.015, 1.018]	13	.54

*Note*: Significant difference between groups (*P* ≤ .05) are highlighted in bold.

Abbreviations: ALP, alkaline phosphatase; ALT, alanine aminotransferase; BCS, body condition score; FGF23, fibroblast growth factor‐23; HCO_3_^−^, bicarbonate; MCS, muscle condition score; n, number of cats; PRD, phosphate‐restricted diet; PTH, parathyroid hormone; SBP, systolic blood pressure; SDMA, symmetric dimethylarginine; USG, urine specific gravity.

^a^ Baseline FGF23 and PTH were log‐transformed for comparison using independent samples *t*‐test and Mann‐Whitney *U* test, respectively.

^b^ Combined PTH results were obtained from immunoradiometric assay (n = 22) and 2‐site immunoenzymatic assay (n = 20).

**TABLE 3:** Descriptive statistics at enrolment for the per‐protocol cats (n = 44) in this prospective dietary trial (MAGMA), grouped based on the allocated study diet (control PRD vs magnesium‐enriched PRD).

Variables (reference interval)	Control PRD (n = 23)		Magnesium‐enriched PRD (n = 21)		*P*‐value
	Median [25th, 75th Percentile]	n	Median [25th, 75th Percentile]	n	
Age (years)	16.2 [13.1, 17.6]	23	14.3 [13.0, 17.2]	21	.63
BCS (“1‐3”, “4‐9”, n [%])	7 [30], 16 [70]	23	4 [21], 15 [79]	19	.73
MCS (“0”, “1”, “2”, “3”, n [%])	0[0], 10 [43], 12 [52], 1 [4]	23	1 [5], 6 [32], 8 [42], 4 [21]	19	.24
Weight (kg)	3.7 [3.2, 4.4]	23	4.2 [3.3, 4.9]	20	.61
Sex (female neutered, n [%])	12 [52]	23	7 [33]	21	.21
Albumin (2.5‐4.5 g/dL)	3.0 [2.8, 3.2]	23	3.0 [2.8, 3.1]	21	.8
ALP (≤60 U/L)	24 [20, 31]	23	27 [19, 32]	21	.71
ALT (5‐60 U/L)	54 [46, 65]	23	46 [36, 63]	21	.11
Chloride (100‐124 mEq/L)	117 [114, 117]	12	117 [116, 119]	13	.75
Creatinine (0.23‐2 mg/dL)	2.41 [1.96, 2.81]	23	2.39 [2.16, 2.84]	21	.63
FGF23^a^ (56‐700 pg/mL)	434 [230, 814]	22	423 [242, 678]	21	.74
Glucose (54‐117 mg/dL)	117 [99, 132]	20	114 [101, 128]	19	.91
Venous HCO_3_^−^ (17‐24 mEq/L)	21.9 [18.6, 23.3]	19	21.4 [19.9, 22.2]	18	.69
Hypertension (controlled) (n [%])	5 [22]	23	4 [19]	21	1
Ionized calcium (4.76‐5.48 mg/dL)	5.36 [5.2, 5.56]	19	5.28 [5.16, 5.36]	18	.45
PCV (30%‐45%)	34 [31, 36]	23	34 [30, 36]	21	.38
Venous pH (7.21‐7.44)	7.38 [7.34, 7.39]	19	7.38 [7.35, 7.39]	18	.93
Phosphate (2.79‐6.81 mg/dL)	3.99 [3.25, 4.40]	23	3.50 [3.28, 3.84]	21	.18
Potassium (3.5‐5.5 mEq/L)	3.94 [3.63, 4.21]	13	4.01 [3.63, 4.32]	13	.62
PTH^a^, ^b^ (2.6‐17.6 pg/mL)	10 [7.2, 18]	17	14.7 [6.9, 22.3]	16	.36
SBP (<160 mmHg)	132 [122, 140]	23	137 [126, 148]	21	.45
SDMA (1‐14 μg/dL)	18 [15, 21]	23	18 [15, 20]	21	.83
Sodium (145‐157 mEq/L)	153 [152, 155]	13	153 [151, 155]	13	.71
Total calcium (8.2‐11.8 mg/dL)	10.32 [9.88, 10.88]	23	10.12 [9.88, 10.44]	21	.2
Total magnesium (1.73‐2.57 mg/dL)	2.09 [1.87, 2.19]	23	2.04 [1.87, 2.19]	21	.77
Total protein (6.0‐8.0 g/dL)	8.0 [7.4, 8.3]	23	7.7 [7.3, 8.2]	21	.56
Urea (7.0‐27.7 mg/dL)	47.3 [44.8, 51.8]	13	47.3 [40.9, 56.6]	13	.55
USG (≥1.035)	1.017 [1.013, 1.018]	13	1.015 [1.015, 1.018]	9	.97

Abbreviations: ALP, alkaline phosphatase; ALT, alanine aminotransferase; BCS, body condition score; FGF23, fibroblast growth factor‐23; HCO_3_^–^, bicarbonate; MCS, muscle condition score; n, number of cats; PRD, phosphate‐restricted diet; PTH, parathyroid hormone; SBP, systolic blood pressure; SDMA, symmetric dimethylarginine; USG, urine specific gravity.

^a^ Baseline FGF23 and PTH were log‐transformed for comparison using independent samples *t*‐test.

^b^ Combined PTH results were obtained from immunoradiometric assay (n = 19) and 2‐site immunoenzymatic assay (n = 14).

All enrolled cats had been eating ≥50% of a standardized PRD before recruitment, for a median of 101 (42, 355) days at enrollment. No difference was found in the period of dietary phosphate restriction between the magnesium and control groups, with a median of 62 (44, 343) days and 112 (42, 371) days, respectively (*P* = .73).

### 3.2 Correlations between baseline total magnesium and CKD‐MBD variables

Correlation analysis showed that baseline plasma tMg correlated negatively with FGF23 (*r* = −0.29; *P* = .03) and positively with plasma creatinine concentration (*r*_*s*_ = 0.34; *P* = .01). Plasma creatinine concentration correlated positively with FGF23 (*r*_*s*_ = 0.32; *P* = .02). No significant correlations were found between tMg and tCa, iCa, SDMA, urea, phosphate, or PTH (Table 4).

**TABLE 4:** Correlations with total magnesium at enrolment for all randomized cats (n = 60) in this prospective dietary trial (MAGMA).

Variables	*r*	*P*‐value
Creatinine (mg/dL)	0.34	**.01**
FGF23 (pg/mL)	(−0.29)	**.03**
Ionized calcium (mg/dL)	0	.99
Phosphate (mg/dL)	0.11	.4
PTH^a^ (pg/mL)	0.13	.4
SDMA (μg/dL)	0.13	.31
Total calcium (mg/dL)	0.04	.78
Urea (mg/dL)	0.19	.28

*Note*: Significant correlations (*P* ≤ .05) are highlighted in bold.

Abbreviations: FGF23, fibroblast growth factor‐23; PTH, parathyroid hormone; *r*, correlation; SDMA, symmetric dimethylarginine.

^*a*^ Combined PTH results were obtained from immunoradiometric assay (n = 22) and 2‐site immunoenzymatic assay (n = 20).

### 3.3 Changes in clinical variables over time in relation to dietary magnesium supplementation

#### 3.3.1 Intention‐to‐treat analysis

**FIGURE 2:**
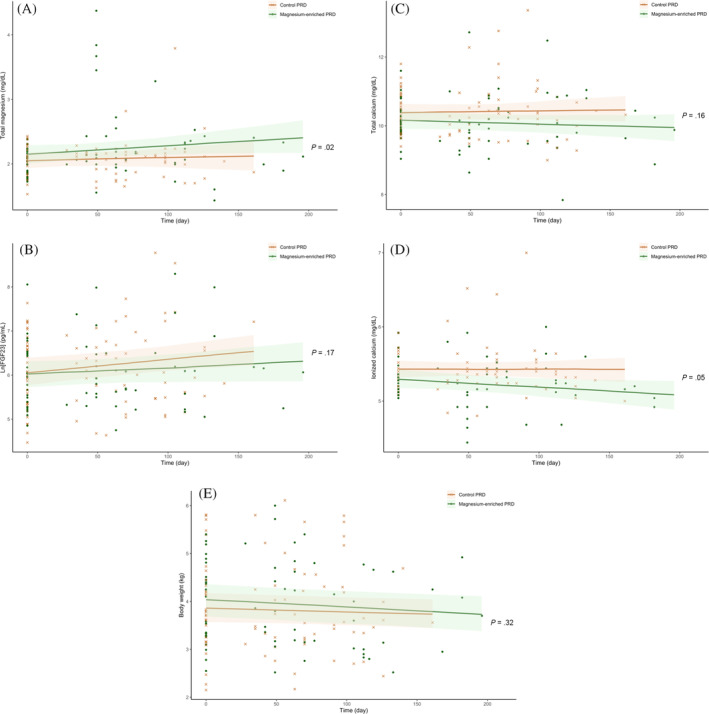
Scatter plots illustrating the linear change of plasma concentrations of (A) total magnesium (tMg); (B) log‐transformed fibroblast growth factor‐23 (ln[FGF23]); (C) total calcium (tCa); (D) ionized calcium (iCa); and (E) body weight in all randomized cats (n = 60) according to the allocated trial diet (“control phosphate‐restricted diet [PRD]” [crosses] vs “magnesium‐enriched PRD” [dots]) during the study period. The *P*‐value refers to the Group × Time interaction (as shown in Table 5) analyzed using linear mixed effects models, which assessed the difference in rate of change of the outcome variable between groups (“control PRD” vs “magnesium‐enriched PRD”) over time.

**FIGURE 3:**
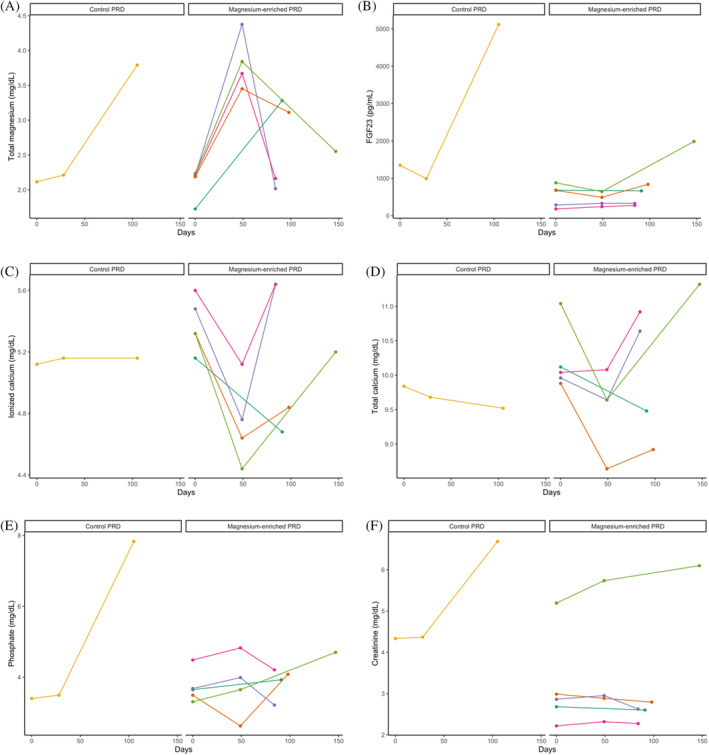
Line graphs illustrating the change of plasma concentrations of (A) total magnesium (tMg); (B) fibroblast growth factor‐23 (FGF23); (C) ionized calcium (iCa); (D) total calcium (tCa); (E) phosphate; and (F) creatinine in individual cats with chronic kidney disease (CKD) that developed hypermagnesemia (n = 6) grouped according to allocation of trial diet (“control phosphate‐restricted diet [PRD]” vs “magnesium‐enriched PRD”) during the study period of this prospective diet trial (MAGMA).

A summary of the results from intention‐to‐treat (n = 60) are presented in Table 5 and Table S4. Rate of change in plasma tMg differed significantly between groups (*P* = .02; Figure 2A); only cats fed a magnesium‐enriched PRD had significantly increased plasma tMg over time (β, 0.17 ± .05 mg/dL/month; *P* < .001) with no change for those fed control PRD. Six cats (5 eating the magnesium‐enriched PRD and 1 eating control PRD) reached trial termination because of the development of moderate hypermagnesemia, with a median plasma tMg of 3.74 (range, 3.28‐4.37) mg/dL, at a median of 49 (range, 49‐105) days after starting the trial (Figure 3). One cat from each group had severe azotemia with plasma creatinine concentration of 5.74 and 6.69 mg/dL, respectively, at the time of hypermagnesemia. All 6 cats were reported to have eaten the trial diets exclusively since enrollment.

**TABLE 5:** Linear mixed model, generalized estimating equation and generalized linear mixed model analyses examining the change in clinicopathological variables over time in all randomized cats (n = 60) during the study period.

Variables	Group	Time	Group × Time
BCS^a^ (“1‐3”, “4‐9”)	.52	.21	.82
MCS^a^ (“0”, “1”, “2”, “3”)	.15	.24	.79
Body weight (kg)	.5	**.002**	.32
Albumin (g/dL)	.89	.2	.78
ALP (U/L)	.85	.52	.14
ALT^a^ (U/L)	.3	.87	.4
Chloride (mEq/L)	.92	.23	.41
Creatinine (mg/dL)	.97	.75	.79
ln[FGF23] (pg/mL)	.88	**.01**	.17
Glucose (mg/dL)	.89	.18	.63
Venous HCO_3_^−a^ (mEq/L)	.82	.66	.35
Ionized calcium (mg/dL)	.21	.37	.052
PCV (%)	.64	.12	.71
Venous pH	.17	.88	.53
Phosphate (mg/dL)	.15	**.04**	.71
Potassium (mEq/L)	.28	.4	.82
ln[PTH]^a^ (pg/mL)	.35	.77	.4
SBP (mmHg)	.92	.6	.68
SDMA (μg/dL)	.64	.14	.96
Sodium (mEq/L)	.4	.84	.14
Total calcium (mg/dL)	.26	.82	.16
Total magnesium (mg/dL)	1	**.01**	**.02**
Total protein (g/dL)	.55	.23	.64
Urea (mg/dL)	.78	.45	.29

*Note*: Summary of *P*‐values for all variables included in the model. Group represents cats in “control PRD” or “magnesium‐enriched PRD” group based on the allocated trial diet according to the randomization list. Outcome variables showing significant change over time and between groups (*P* ≤ .05) are highlighted in bold. The unit used for time was month (30.4 days). A significant effect in the group column indicates a significant difference between the 2 groups at baseline for a given parameter (the start of the regression line at time 0). A significant effect of Group × Time interaction indicates that the rate of change of the outcome variable differs significantly between groups (“control PRD” vs “magnesium‐enriched PRD”) over time. If Group × Time was not significant, and the effect of Time was significant, this indicates no difference in the rate of change of the outcome variable between groups, however, the overall gradient of the outcome variable plotted against time (with the data from all groups combined) differs significantly from zero.

Abbreviations: ALP, alkaline phosphatase; ALT, alanine aminotransferase; BCS, body condition score; HCO_3_^−^, bicarbonate; ln[FGF23], log‐transformed fibroblast growth factor‐23; ln[PTH], log‐transformed parathyroid hormone; MCS, muscle condition score; SBP, systolic blood pressure; SDMA, symmetric dimethylarginine.

^a^ Only the case number of each individual cat was included as random effect in the model.

Although no difference was found in the rate of change in plasma phosphate concentration (*P* = .71) and ln[FGF23] (*P* = .17) between groups, cats fed a control PRD had a significant increase in ln[FGF23] (β, 0.12 ± .04 pg/mL/month; *P* = .003), but ln[FGF23] remained stable over time in the magnesium group (Figure 2B). Rate of change in blood iCa differed, albeit not significantly (*P* = .05), between groups, with decreasing and increasing iCa observed in cats fed a magnesium‐enriched and control PRD, respectively (Figure 2D). A significantly lower proportion of magnesium supplemented CKD cats had “uptrend” iCa during the trial than those in the control group (9.1% vs 40%; *P* = .02). At the end of the trial, blood iCa was lower in cats fed a magnesium‐enriched PRD than in those fed a control PRD (5.2 [4.96, 5.4] vs 5.4 [5.24, 5.56] mg/dL; *P* = .01). Body weight decreased by 0.7% to 1.1% per month in both groups with no difference in rate of change identified between groups (*P* = .62), but the decrease was only significant in the magnesium group (β, 0.046 ± .015 kg/month; *P* = .01; Figure 2E).

#### 3.3.2 Per‐protocol analysis

**FIGURE 4:**
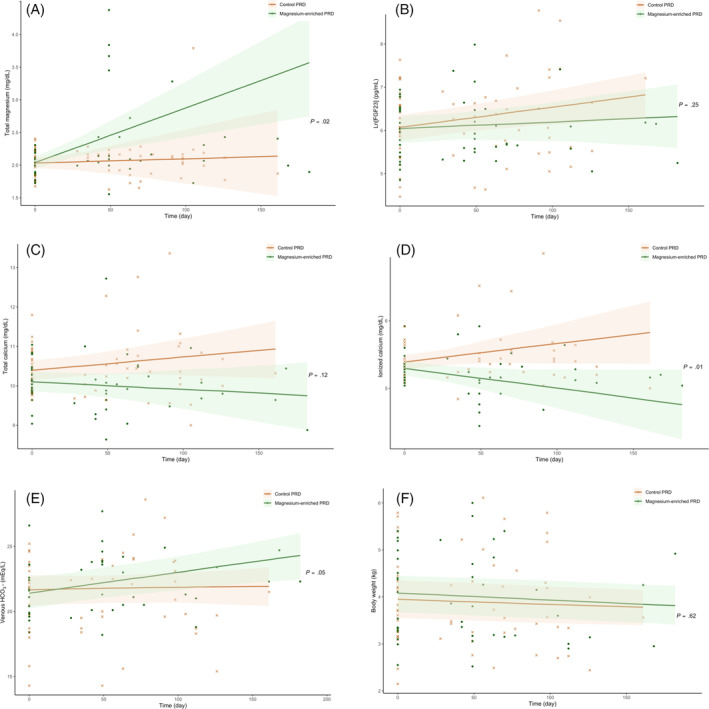
Scatter plots illustrating the linear change of plasma concentrations of (A) total magnesium concentration (tMg); (B) log‐transformed fibroblast growth factor‐23 (ln[FGF23]); (C) total calcium (tCa); (D) ionized calcium (iCa); (E) venous bicarbonate (HCO3^−^); and (F) body weight in the per‐protocol cats (n = 44) according to the allocated trial diet (“control phosphate‐restricted diet [PRD]” [crosses] vs “magnesium‐enriched PRD” [dots]) during the study period. The *P*‐value refers to the Group × Time interaction (as shown in Table 6) analyzed using linear mixed effects models, which assessed the difference in rate of change of the outcome variable between groups (“control PRD” vs “magnesium‐enriched PRD”) over time.

A summary of the results from per‐protocol analysis (n = 44) is presented in Table 6 and Table S5. The results confirmed a significant increase in plasma tMg by an average of 1.2% per month, in magnesium supplemented cats (β, 0.25 ± .07 mg/dL/month; *P* < .001), but no change in the controls (Figure 4A). Plasma ln[FGF23] increased significantly in the control group, by an average of 2.3% per month (β, 0.14 ± .05 pg/mL/month; *P* = .01), but remained stable in the magnesium group (β, 0.05 ± .06 pg/mL/month; *P* = .37; Figure 4B). Rate of change in blood iCa differed significantly between groups (*P* = .01), with decreasing and increasing iCa observed in cats fed a magnesium‐enriched PRD and a control PRD, respectively (Figure 4D). Similar to the intention‐to‐treat analysis, a lower proportion of magnesium supplemented cats had “uptrend” iCa compared to those eating a control PRD (11.8% vs 52.9%; *P* = .03). At the end of the trial, both iCa and tCa were significantly lower in cats fed a magnesium‐enriched PRD than in those fed a control PRD (iCa, 5.12 [4.92, 5.24] vs 5.48 [5.24, 5.68] mg/dL); *P* < .001; tCa, 9.8 (9.48, 10.44) vs 10.36 (10.04, 11.04) mg/dL; *P* = .01. Venous HCO_3_^−^ concentration also increased significantly, by an average of 2.4% per month, in cats eating a magnesium‐enriched PRD (β, 0.51 ± .16 mEq/L/month; *P* = .004), but no change was observed in cats eating a control PRD (Figure 4E). Body weight decreased significantly in both groups, with an average decrease of 1.1% and 0.8% per month in the magnesium group (β, −0.04 ± .02 kg per month; *P* = .01) and control group (β, −0.03 ± .02 kg per month; *P* = .05), respectively (Figure 4F); no difference in the rate of change was found between groups (*P* = .62).

**TABLE 6:** Linear mixed model, generalized estimating equation, and generalized linear mixed model analyses examining the change in clinicopathological variables over time in the per‐protocol cats (n = 44) during the study period.

Variables	Group	Time	Group × Time
BCS^a^ (“1‐3”, “4‐9”)	.75	.08	.66
MCS^a^ (“0”, “1”, “2”, “3”)	.51	.17	.87
Body weight^a^ (kg)	.6	**.002**	.62
Albumin (g/dL)	.71	.74	.77
ALP (U/L)	.68	.51	.29
ALT^a^ (U/L)	.57	.623	.4
Chloride (mEq/L)	.64	.1	.65
Creatinine (mg/dL)	.6	.73	.68
Ln[FGF23] (pg/mL)	.84	**.02**	.25
Glucose (mg/dL)	.99	.19	.28
Venous HCO_3_^−^ (mEq/L)	.76	**.01**	**.05**
Ionized calcium (mg/dL)	.21	.91	**.01**
PCV (%)	.3	.21	.73
Venous pH^a^	.57	.47	.31
Phosphate (mg/dL)	.37	.26	.55
Potassium (mEq/L)	.56	.64	.71
Ln[PTH] (pg/mL)	.14	.93	.49
SBP (mmHg)	.64	.83	.91
SDMA (μg/dL)	.61	.39	.78
Sodium^a^ (mEq/L)	.32	.98	.16
Total calcium (mg/dL)	.21	.91	**.01**
Total magnesium (mg/dL)	.88	**.01**	**.02**
Total protein (g/dL)	.81	.75	.96
Urea (mg/dL)	.18	.71	.44

*Note*: Summary of *P*‐values for all variables included in the model. Group represents cats in “control phosphate‐restricted diet (PRD)” or “magnesium‐enriched PRD” group based on the allocated trial diet according to the randomization list. Outcome variables showing significant change over time and between groups (*P* ≤ .05) are highlighted in bold. The unit used for time was month (30.4 days). A significant effect in the group column indicates a significant difference between the 2 groups at baseline for a given parameter (the start of the regression line at time 0). A significant effect of Group × Time interaction indicates that the rate of change of the outcome variable differs significantly between groups (“control PRD” vs “magnesium‐enriched PRD”) over time. If Group × Time was not significant, and the effect of Time was significant, this indicates no difference in the rate of change of the outcome variable between groups; however, the overall gradient of the outcome variable plotted against time (with the data from all groups combined) differs significantly from zero.

Abbreviations: ALP, alkaline phosphatase; ALT, alanine aminotransferase; BCS, body condition score; HCO_3_^−^, bicarbonate; ln[FGF23], log‐transformed fibroblast growth factor‐23; ln[PTH], log‐transformed parathyroid hormone; MCS, muscle condition score; SBP, systolic blood pressure; SDMA, symmetric dimethylarginine.

^a^ Only the case number of each individual cat was included as random effect in the model.

#### 3.3.3 Clinical events

Clinical events that occurred during the trial are summarized in Table 7. Both trial diets were well tolerated without substantive adverse effects. Overall, 3 cats (all in the control group) were euthanized because of renal disease (n = 2) or liver disease (n = 1). Two control group cats developed diabetes mellitus and 1 cat from the magnesium group had a suspected intestinal mass palpated at its 1st follow‐up revisit. During the trial period, 6 cats developed hypermagnesemia, 5 of which were magnesium supplemented (Figure 3). None of the cats that developed hypermagnesemia had adverse effects observed, with no evidence of either diarrhea or crystalluria. However, in all cases, the trial diet was discontinued and cats were transitioned back onto their original commercial PRD. Four of the 6 cats developed hypermagnesemia after 49 days at their 1st follow‐up visit (all in the magnesium group); plasma tMg in these cats increased by an average of 73% (range, 58%‐98%), whereas iCa decreased by an average of 13% (range, 9%‐17%). Their iCa increased by an average of 12.5% (range, 4%‐18%) after being transferred back to their original commercial PRD (Figure 3).

**TABLE 7:** Clinical events in all randomized cats (n = 60) during this prospective dietary trial (MAGMA).

Clinical event	Control PRD (n = 33)	Magnesium‐enriched PRD (n = 27)
Deaths	3 (9%)	0
	Renal disease (n = 2)	…
	Liver disease (n = 1)	…
Comorbidities	2 (6%)	1 (4%)
	Diabetes mellitus (n = 2)	Suspected intestinal mass (n = 1)
Adverse events	10 (30%)	7 (26%)
	Hypermagnesemia^a^ (n = 1)	Hypermagnesemia^a^ (n = 5)
	Intermittent diarrhea (n = 5)	Intermittent diarrhea (n = 2)
	Hypercalcemia^b^ (n = 4)	…

Abbreviation: PRD, phosphate‐restricted diet.

^a^ Hypermagnesemia was defined as plasma total magnesium concentration (tMg) >2.92 mg/dL (1.2 mmol/L).

^b^ Hypercalcemia was defined as blood ionized calcium (iCa) concentration >6 mg/dL (1.5 mmol/L).

Seven cats developed intermittent diarrhea (2 in the magnesium group, 5 in the control group). Four cats from the control group developed ionized hypercalcemia during the trial and were transitioned onto a moderately PRD (Feline Veterinary Early Renal [dry and wet], Royal Canin SAS, Aimargues, France) with a phosphorus content of 1.5 g/Mcal and Ca : P of 1.3.

## 4 DISCUSSION

Our study is the 1st randomized controlled clinical trial to evaluate the effect of short‐term dietary magnesium supplementation on CKD‐MBD in cats. Feeding a magnesium supplemented PRD for 4 months substantially increased plasma tMg (without hypermagnesemia in most cases) and venous HCO_3_^−^ concentration. Cats with CKD eating a magnesium‐enriched PRD also exhibited greater stability in plasma FGF23 and iCa over time compared with the CKD cats fed a control PRD, indicating a potential benefit of dietary magnesium supplementation on the control of CKD‐MBD in cats with low or normal tMg. These beneficial effects of dietary magnesium supplementation were of a clinically relevant magnitude.

In cats with CKD eating ≥50% of a magnesium‐enriched PRD, dietary supplementation of MgO significantly increased plasma tMg over time, with an average increment of 12% per month. This finding is in agreement with numerous studies showing an increase in serum magnesium concentration after PO magnesium supplementation in rodent models^23^, ^24^ and human CKD patients.^25^, ^26^, ^27^, ^28^ Intestinal magnesium absorption correlates positively with dietary magnesium intake.^29^, ^30^ In our study, 5 cats (23.8%) in the magnesium group developed moderate hypermagnesemia and were removed from the trial, compared with only 1 cat (4.3%) in the control group (Figure 3). The control cat that developed moderate hypermagnesemia had progressive azotemia and was euthanized 3 days after the end of the trial; plasma tMg and plasma creatinine concentration were increased by 79.3% and 54.3% within 15 weeks, respectively. This finding suggested that, unlike the cats fed a magnesium‐enriched PRD, accumulation of magnesium in this cat likely was caused by decreased urinary clearance of magnesium. A recent retrospective study found that most IRIS stage 4 CKD cats were hypermagnesemic.^5^

The rate of change in iCa differed significantly between the 2 study groups in per‐protocol analysis, with decreasing and increasing trends observed in cats fed a magnesium‐enriched PRD and control PRD, respectively. Magnesium supplemented cats were less likely to develop an increasing trend in iCa compared to those fed a control PRD; 4 cats from the control group developed ionized hypercalcemia (iCa >6 mg/dL), whereas none in the magnesium group did. Four cats in the magnesium group developed moderate hypermagnesemia at their 1st follow‐up visits after eating exclusively a magnesium‐enriched PRD for 7 weeks. A concomitant decrease in tMg and increase in iCa were observed after transferring them onto the original standardized PRD (Figure 3), reinforcing the calcium‐lowering effect of magnesium. In parallel, a meta‐analysis of 3 randomized controlled trials^26^, ^27^, ^31^ recently has shown that dietary magnesium supplementation significantly decreased serum calcium concentration in human hemodialysis patients,^14^ supporting our results and the comparable effect of dietary magnesium on calcium control between human patients and cats with CKD. The calcium‐lowering effect associated with the additional MgO in the magnesium‐enriched PRD remains to be determined. It could be explained by a decrease in intestinal absorption of calcium.^32^, ^33^, ^34^ However, conflicting data exist on the relationship between magnesium intake and calcium balance. Some studies showed that dietary magnesium supplementation increased intestinal calcium absorption,^35^, ^36^, ^37^ whereas others found no effect.^38^ Another explanation could be attributed to enhanced urinary excretion of calcium induced by magnesium, possibly caused by the antagonism of magnesium ions as they compete for the same paracellular route for renal reabsorption,^39^ and inhibition of calcium uptake via transient receptor potential vanilloid‐5 in distal convoluted tubules by magnesium.^40^ The exact mechanisms underlying the effect of magnesium on calcium in cats with CKD remain to be determined.

Dietary phosphate restriction is the mainstay of CKD management in cats, but feeding a PRD may be associated with development of hypercalcemia in some cats.^41^, ^42^, ^43^, ^44^ Moderate dietary phosphorus attenuation normalized calcium status in these cats, which is likely, at least in part, attributed to the decrease in dietary Ca : P, and no apparent changes in kidney function were observed in the study.^42^ This hypothesis is further supported by a recent case series that reported the calcium‐lowering effect of diets with a calcium content of <2 g/Mcal and Ca : P of <1.4 in successfully managing idiopathic or CKD‐associated hypercalcemia in cats.^45^ However,^41^ long‐term implications on renal health and mortality associated with the consumption of a less PRD in azotemic CKD cats have not been investigated. Increasing plasma calcium concentration in cats with CKD was independently associated with nephrocalcinosis and progression of azotemia.^46^, ^47^ Therefore, the calcium‐stabilizing effect of a magnesium‐enriched PRD may assist in countering the hypercalcemic effect of PRDs, without compromising the degree of dietary phosphate restriction, in certain cats with CKD. Future prospective randomized clinical trials comparing long‐term implications of a moderately phosphate‐restricted diet with magnesium‐enriched PRD in azotemic CKD cats with hypercalcemia are warranted.

Significant reductions in plasma FGF23, plasma phosphate concentration and plasma PTH concentration were observed in CKD cats fed a commercially available PRD for 4 to 8 weeks.^48^ All cats in our study were fed a standardized PRD for a median of 14 weeks before commencement of the randomized diet trial, which ensured that the CKD‐MBD variables from all enrolled cats were stabilized and therefore any clinicopathological changes identified during the trial were primarily attributed to the alteration made in the treatment (ie, dietary magnesium content).

Plasma FGF23 concentration was found to significantly increase in cats fed a control PRD, whereas it did not change in those fed a magnesium‐enriched PRD. Our finding suggests that dietary magnesium supplementation could further stabilize FGF23 in cats with azotemic CKD that have already been transitioned to a PRD. Because excess FGF23 concentration is known to be associated with phosphate disturbance and progression of CKD in cats,^15^, ^20^ a magnesium‐enriched PRD may provide additional benefit in phosphate homeostasis. This hypothesis is supported by the phosphate‐binding ability of magnesium.^26^ Consistent results regarding FGF23 suppression by MgO have been reported in human patients undergoing hemodialysis.^13^ Dietary loading of magnesium also has been shown to decrease serum FGF23 concentration in rodent models with normal kidney function.^12^ Although a decrease in plasma FGF23 concentration was not observed in the cats fed a magnesium‐enriched PRD, it did increase significantly in those maintained on a control PRD. A negative correlation also was found between baseline plasma tMg and FGF23 concentration in all randomized cats in our study (Table 4), emphasizing the potential involvement of magnesium in FGF23 regulation.

Bicarbonate is a base buffer that helps to prevent metabolic acidosis, a common complication associated with CKD, which contributes to disease progression.^49^ Our results showed that CKD cats fed a magnesium‐enriched PRD had increased venous HCO_3_^−^ concentration over time, suggesting that dietary magnesium supplementation with MgO may prevent development of metabolic acidosis and further stabilize CKD in cats. Increased serum HCO_3_^−^ concentration is associated with decreased risk of CKD progression and all‐cause mortality in human patients with CKD.^50^ Meta‐analysis also demonstrated the effectiveness of PO sodium bicarbonate in increasing serum HCO_3_^−^ concentration and delaying CKD progression in human patients.^51^ Metabolic acidosis is associated with increased bone resorption and decreased bone formation^52^; increased venous HCO_3_^−^ concentration could be responsible for the decrease in plasma iCa observed in our study caused by a decrease in calcium mobilization from bone.^53^ Additional studies are warranted to determine the effects of magnesium supplementation on bone turnover.

Body weight of enrolled cats decreased over the study period, with no difference in rate of change between the 2 diet groups. These results agree with previous findings from several cohort studies showing a significant decrease in body weight in CKD cats over time.^46^, ^47^, ^54^ Sixteen of 60 (26.7%) cats enrolled in our trial did not have a follow‐up visit with ≥50% of trial diet consumed during the trial. Of the 16 cats that were excluded from the per‐protocol analysis, 6 cats (22.2%) were allocated to the magnesium group whereas 10 cats (30.3%) were allocated to the control group with no difference in dietary adherence observed between groups (*P* = .48). Overall, no difference in acceptability was found between trial diets. Although 5 magnesium supplemented CKD cats developed hypermagnesemia, none of them had any adverse effects observed throughout the trial.

Our clinical trial had several limitations. Protocol amendment to increase the inclusion limit of plasma tMg was made during the study because of the low prevalence (12%) of CKD cats with hypomagnesemia reported in a cohort study.^5^ Subanalyses were performed to compare baseline characteristics between the 2 treatment groups before and after the amendment; results similar to the main findings (Table 2) were obtained, supporting the similarity in cats recruited and therefore pooling of all enrolled cats for analyses was deemed admissible.^55^ Sixteen cats (26.7%) were excluded in our per‐protocol analysis because of dietary nonadherence (<50% of trial diet consumed as defined a priori). A similar proportion of cats was excluded from each diet group, suggesting that the palatability of the 2 study diets was comparable, with no food aversion toward the test diet and therefore minimizing the possibility of attrition bias. An adherence of 73% for a dietary interventional trial is considered acceptable.^56^

Lack of adequate masking of intervention remains a major challenging issue in randomized dietary clinical trials^57^ and was a possibility during our study because 5 cats developed hypermagnesemia in 1 arm of the trial, compared with only 1 cat in the other arm. The impact of this difference was minimized by focusing the study results on measurements of objectively quantifiable variables (eg, FGF23, tCa) that were measured in a blinded fashion.

In conclusion, our results showed that dietary magnesium supplementation was successful in increasing tMg in cats with CKD. Additionally, our study provided clear evidence that a magnesium‐enriched PRD stabilizes plasma calcium and FGF23 concentrations in CKD cats with low or normal plasma tMg, compared with their counterparts eating a standardized PRD. These findings may provide a foundation for future therapeutic strategies in the management of hypercalcemia in cats with CKD, especially for cats that develop hypercalcemia secondary to dietary phosphate restriction. However, before dietary magnesium supplementation can be recommended as part of the armamentarium to better control CKD‐MBD in cats, future studies with a longer follow‐up period will be required to evaluate not only long‐term effects on magnesium status but also effects on the progression of CKD and survival outcome in cats with CKD.

## CONFLICT OF INTEREST DECLARATION

Pak‐Kan Tang received a PhD studentship funded by Royal Canin SAS. Rebecca Geddes received funding from Petplan, Royal Canin, an RVC Internal Grant, The Academy of Medical Sciences and The Everycat Foundation; has previously had a consultancy agreement with Boehringer Ingelheim; has received speaking honoraria from Boehringer Ingelheim, Idexx and Royal Canin. Rosanne Jepson received funding from PetPlan, Feline Foundation for Renal Research, RVC Internal Grant, PetSavers, and consultancy agreements: Boehringer Ingelheim, Merial, CEVA. Speaking honoraria: Boehringer Ingelheim, Hills Pet Nutrition, CEVA. Jonathan Elliott has Consultancy agreements with: Elanco Ltd, CEVA Animal Health Ltd, Boehringer Ingelheim Ltd, MSD Animal Health Ltd, Orion Incorp, Idexx Ltd, Waltham Petcare Science Institute, Invetx Inc and Zoetis Ltd received grant funding from Elanco Ltd, Waltham Centre for Pet Nutrition, Royal Canin SAS, Idexx Ltd, CEVA Animal Health. He is a member of the International Renal Interest Society which receives sponsorship from Zoetis.

## OFF‐LABEL ANTIMICROBIAL DECLARATION

Authors declare no off‐label use of antimicrobials.

## INSTITUTIONAL ANIMAL CARE AND USE COMMITTEE (IACUC) OR OTHER APPROVAL DECLARATION

Approved by the Ethics and Welfare Committee of the Royal Veterinary College (URN 2017 1713‐3).

## HUMAN ETHICS APPROVAL DECLARATION

Authors declare human ethics approval was not needed for this study.

## Supporting information


**Data S1:** Supplementary material.

**Figure S1.** Baseline dietary questionnaire for this prospective dietary trial (MAGMA).

**Figure S2.** Follow‐up dietary questionnaire for this prospective dietary trial (MAGMA).

**Figure S3.** Bland‐Altman plot illustrating the difference between log‐transformed parathyroid hormone (lnPTH) measurements obtained from an immunoradiometric assay (IRA) and a 2‐site immunoenzymatic assay (IEA).

**Table S1.** Participant information sheet for this prospective dietary trial (MAGMA).

**Table S2.** Consent form for this prospective dietary trial (MAGMA).

**Table S3.** Nutritional composition and ingredients for each phosphate‐restricted diet (PRD).

**Table S4.** Linear mixed model, generalized estimating equation and generalized linear mixed model analyses examining the change in clinicopathological variables over time in all randomized cats (n = 60) during the study period. Summary of intercepts and the slopes between groups (“control PRD” or “magnesium‐enriched PRD”).

**Table S5.** Linear mixed model, generalized estimating equation and generalized linear mixed model analyses examining the change in clinicopathological variables over time in the per‐protocol cats (n = 44) during the study period. Summary of intercepts and the slopes between groups (“control PRD” or “magnesium‐enriched PRD”).
