# Supplementary material for: Dietary magnesium supplementation in cats with chronic kidney disease: A prospective double‐blind randomized controlled trial
Source: J Vet Intern Med. 2024 Jul 1;38(4):2180–95. doi: 10.1111/jvim.17134 (PMC11256178; doi:10.1111/jvim.17134)
Supplement: Supplementary file 1 — Data S1: Supplementary material. [file JVIM-38-2180-s005.docx]

Baseline characteristics of the cats with overt hypercalcemia (n = 3).

| **Variables** | **Cat #6** | **Cat #20** | **Cat #21** |
| --- | --- | --- | --- |
| BCS (“1–3”, “4–6”, “7–9”) | 3 | 4 | 4 |
| MCS (“0”, “1”, “2”, “3”) | 1 | 2 | 1 |
| Body weight (kg) | 2.4 | 4.1 | 3.9 |
| Albumin (2.5–4.5 g/dL) | 3.1 | 3.3 | 3.2 |
| ALP (≤ 60 U/L) | 43 | 46 | 21 |
| ALT (5–60 U/L) | 36 | 134 | 78 |
| Chloride (100–124 mEq/L) | NA | 116 | 115 |
| Creatinine (0.23–2 mg/dL) | 2.52 | 1.95 | 2.15 |
| FGF23 (56–700 pg/mL) | 4307 | 1085 | 14818 |
| Glucose (54–117 mg/dL) | 126.1 | NA | 86.5 |
| Venous HCO_3_^–^ (17–24 mEq/L) | 25.4 | NA | 21.8 |
| Ionized calcium (4.76–5.48 mg/dL) | 6.04 | NA | 6.24 |
| PCV (30–45%) | 38 | 38 | 32 |
| Venous pH (7.21–7.44) | 7.388 | NA | 7.405 |
| Phosphate (2.79–6.81 mg/dL) | 4.24 | 3.07 | 5.51 |
| Potassium (3.5–5.5 mEq/L) | 3.5 | NA | 4 |
| PTH (2.6–17.6 pg/mL) | 5.6 | 8.6 | 5.5 |
| SBP (<160 mmHg) | 107 | 129 | 120 |
| SDMA (1–14 μg/dL) | 34 | 12 | 15 |
| Sodium (145–157 mEq/L) | NA | 158 | 154 |
| Total calcium (8.2–11.8 mg/dL) | 12.6 | 12.6 | 12.36 |
| Total magnesium (1.73–2.57 mg/dL) | 1.77 | 1.94 | 2.02 |
| Total protein (6.0–8.0 g/dL) | 8.4 | 8.5 | 7.3 |
| Urea (7.0–27.7 mg/dL) | NA | 28 | 44 |
| USG (≥1.035) | 1.017 | 1.021 | 1.021 |

Abbreviations: BCS, body condition score; MCS, muscle condition score; ALP, alkaline phosphatase; ALT, alanine aminotransferase; FGF23, fibroblast growth factor-23; HCO_3_^–,^ bicarbonate; PCV, packed cell volume; PTH, parathyroid hormone; SBP, systolic blood pressure; SDMA, symmetric dimethylarginine; USG, urine specific gravity.

Changes in clinicopathological variables of the cats with baseline hypercalcemia (n = 3) over time during the study period.

| **Variables** | **Visit** | **Cat #6** | **Cat #20** | **Cat #21** |
| --- | --- | --- | --- | --- |
| Treatment group | NA | Control | Magnesium | Magnesium |
| Duration between baseline and 1^st^ follow-up (days) | NA | 105 | 126 | 84 |
| Proportion of trial diet consumed (%) | NA | 33 | 90 | 100 |
| BCS (1–9) | Baseline | 3 | 4 | 4 |
|  | Follow-up | 2 | 4 | 4 |
| Body weight (kg) | Baseline | 2.4 | 4.1 | 3.9 |
|  | Follow-up | 2.2 | 3.7 | 3.9 |
| Creatinine (mg/dL) | Baseline | 2.52 | 1.95 | 2.15 |
|  | Follow-up | 2.07 | 1.54 | 2.05 |
| FGF23 (pg/mL) | Baseline | 4307 | 1085 | 14818 |
|  | Follow-up | 4473 | 1031 | 13830 |
| Ionized calcium (mg/dL) | Baseline | 6.04 | NA | 6.24 |
|  | Follow-up | NA | NA | 5.28 |
| Phosphate (mmol/L) | Baseline | 4.24 | 3.07 | 5.51 |
|  | Follow-up | 4.61 | 3 | 5.05 |
| PTH (pg/mL) | Baseline | 5.6 | 8.6 | 5.5 |
|  | Follow-up | 5.1 | 5.4 | 7.3 |
| Total calcium (mg/dL) | Baseline | 12.6 | 12.6 | 12.36 |
|  | Follow-up | 13.04 | 10.92 | 10.2 |
| Total magnesium (mg/dL) | Baseline | 1.77 | 1.94 | 2.02 |
|  | Follow-up | 2.14 | 2.24 | 1.85 |

Abbreviations: BCS, body condition score; FGF23, fibroblast growth factor-23; PTH, parathyroid hormone.
