# Supplementary material for: Dietary magnesium supplementation in cats with chronic kidney disease: A prospective double‐blind randomized controlled trial
Source: J Vet Intern Med. 2024 Jul 1;38(4):2180–95. doi: 10.1111/jvim.17134 (PMC11256178; doi:10.1111/jvim.17134)
Supplement: Supplementary file 4 — Figure S3. Bland‐Altman plot illustrating the difference between log‐transformed parathyroid hormone (lnPTH) measurements obtained from an immunoradiometric assay (IRA) and a 2‐site immunoenzymatic assay (IEA). [file JVIM-38-2180-s001.docx]

**SUPPLEMENTARY FIGURE 3.** Bland-Altman plot illustrating the difference between log-transformed parathyroid hormone (lnPTH) measurements obtained from an immunoradiometric assay (IRA) and a two-site immunoenzymatic assay (IEA).


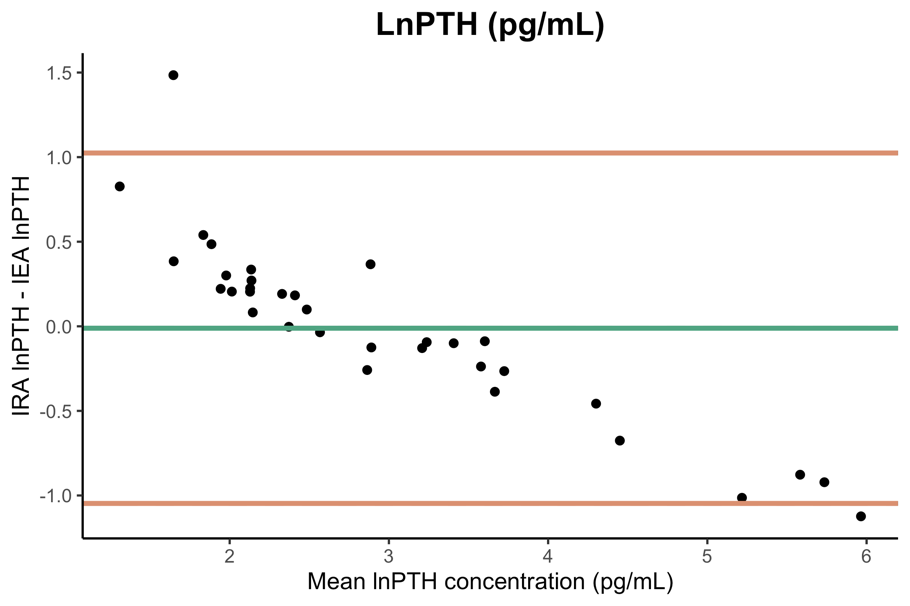


Green lines represent mean differences between methods; orange lines represent limits of agreement.

Abbreviations: IEA, immunoenzymatic assay; IRA, immunoradiometric assay; lnPTH, log-transformed parathyroid hormone.

Thirty-four feline EDTA plasma samples were selected for the comparison of parathyroid hormone (PTH) measurements obtained from an immunoradiometric assay (IRA) and a two-site immunoenzymatic assay (IEA). Both assays had previously been validated to measure plasma PTH in cats. The median plasma PTH concentrations obtained from IRA and IEA were 12.7 [9.33, 32.1] and 12.34 [7.2, 39.8] pg/mL, respectively. Wilcoxon signed-rank test identified no significant difference in PTH measurements between the two assays (*P* = 0.16). An excellent agreement in plasma PTH concentrations, especially when PTH concentrations are within the physiological levels, is illustrated in the Bland-Altman plot. However, plasma PTH concentrations obtained from the IRA appear to be consistently higher than those obtained from the IEA in cats with marked PTH excess.
