# Supplementary material for: Dietary magnesium supplementation in cats with chronic kidney disease: A prospective double‐blind randomized controlled trial
Source: J Vet Intern Med. 2024 Jul 1;38(4):2180–95. doi: 10.1111/jvim.17134 (PMC11256178; doi:10.1111/jvim.17134)
Supplement: Supplementary file 6 — Table S2. Consent form for this prospective dietary trial (MAGMA). [file JVIM-38-2180-s008.docx]

**SUPPLEMENTARY TABLE 2.** Consent form for this prospective dietary trial (MAGMA).
