# Supplementary material for: Dietary magnesium supplementation in cats with chronic kidney disease: A prospective double‐blind randomized controlled trial
Source: J Vet Intern Med. 2024 Jul 1;38(4):2180–95. doi: 10.1111/jvim.17134 (PMC11256178; doi:10.1111/jvim.17134)
Supplement: Supplementary file 7 — Table S3. Nutritional composition and ingredients for each phosphate‐restricted diet (PRD). [file JVIM-38-2180-s003.docx]

**SUPPLEMENTARY TABLE 3.** Nutritional composition and ingredients for each phosphate-restricted diet (PRD).

| **Nutrient** | **Dry formulation^[[1]](#footnote-1)^** | | |  | **Wet formulation^[[2]](#footnote-2)^** | | | |
| --- | --- | --- | --- | --- | --- | --- | --- | --- |
|  | **Renal** | **Renal Special** | **Renal Select** |  | **Chicken** | **Fish** | **Beef** | **Loaf** |
| Moisture (g/Mcal) | 14.01 | 14.03 | 13.47 |  | 654.21 | 842.11 | 796.81 | 740.74 |
| Protein (g/Mcal) | 58.6 | 66.34 | 60 |  | 66.27 | 69.47 | 66.73 | 71.23 |
| Fat content (g/Mcal) | 43.31 | 43.38 | 52.66 |  | 67.97 | 63.16 | 65.74 | 61.73 |
| Crude fibre (g/Mcal) | 11.72 | 12.25 | 11.27 |  | 4.25 | 10.53 | 4.98 | 8.55 |
| Crush ash (g/Mcal) | 15.05 | 15.82 | 16.16 |  | 11.05 | 11.58 | 10.96 | 11.4 |
| Carbohydrate [NFE] (g/Mcal) | 112.36 | 103.34 | 91.35 |  | 45.88 | 55.79 | 50.8 | 56.03 |
| Dietary fibre (g/Mcal) | 27.29 | 27.3 | 23.76 |  | 6.8 | 15.79 | 7.97 | 20.89 |
| Omega 6 (g/Mcal) | 8.54 | 8.32 | 8.84 |  | 17.84 | 14.74 | 23.9 | 10.45 |
| Omega 3 (g/Mcal) | 2.45 | 2.12 | 2.13 |  | 2.38 | 2.74 | 2.9 | 2.85 |
| EPA + DHA (g/Mcal) | 1.07 | 1.05 | 1 |  | 1.33 | 1.31 | 1.35 | 1.28 |
| Calcium (g/Mcal) | 1.53 | 1.61 | 1.71 |  | 1.27 | 1.47 | 1.29 | 1.42 |
| Phosphorus (g/Mcal) | 0.76 | 1.15 | 1 |  | 0.76 | 0.95 | 0.75 | 0.95 |
| Ca:P | 2.01 | 1.4 | 1.71 |  | 1.67 | 1.55 | 1.72 | 1.49 |
| Sodium (g/Mcal) | 1.02 | 1.02 | 1.1 |  | 0.93 | 0.84 | 0.8 | 0.85 |
| Magnesium (g/Mcal) | 0.18 | 0.13 | 0.2 |  | 0.14 | 0.17 | 0.16 | 0.14 |
| Potassium (g/Mcal) | 2.3 | 2.3 | 2.2 |  | 1.87 | 1.89 | 1.89 | 1.9 |
| Vitamin D (IU/Mcal) | 204.3 | 229.65 | 220.43 |  | 365.34 | 347.37 | 418.33 | 360.87 |
| Metabolizable energy^a^ (kcal/kg) | 3921 | 3919 | 4083 |  | 1177 | 950 | 1004 | 1053 |

*^a^Calculated according to the National Research Council (NRC) 2006 equation using crude fibre.*

Abbreviations: Ca:P, calcium-to-phosphorus ratio; DHA, docosahexaenoic acid; EPA, eicosapentaenoic acid; NFE, nitrogen-free extract.

1. Feline Veterinary Diet Renal [dry], Royal Canin SAS, Aimargues, France [↑](#footnote-ref-1)
2. Feline Veterinary Diet Renal [wet], Masterfoods, Bruck, Austria [↑](#footnote-ref-2)
