# Supplementary material for: Dietary magnesium supplementation in cats with chronic kidney disease: A prospective double‐blind randomized controlled trial
Source: J Vet Intern Med. 2024 Jul 1;38(4):2180–95. doi: 10.1111/jvim.17134 (PMC11256178; doi:10.1111/jvim.17134)
Supplement: Supplementary file 8 — Table S4. Linear mixed model, generalized estimating equation and generalized linear mixed model analyses examining the change in clinicopathological variables over time in all randomized cats (n = 60) during the study period. Summary of intercepts and the slopes between groups (“control PRD” or “magnesium‐enriched PRD”). [file JVIM-38-2180-s007.docx]

**SUPPLEMENTARY TABLE 4.** Linear mixed model, generalized estimating equation and generalized linear mixed model analyses examining the change in clinicopathological variables over time in all randomized cats (n = 60) during the study period. Summary of intercepts and the slopes between groups (“control PRD” or “magnesium-enriched PRD”).

| **Variables** | **Control PRD (n = 33)** | | |  | **Magnesium-enriched PRD (n = 27)** | | |
| --- | --- | --- | --- | --- | --- | --- | --- |
|  | Intercept | Coefficient of time  (β; month) | *P*-value |  | Intercept | Coefficient of time  (β; month) | *P*-value |
| BCS^a,b^ (“1–3”, “4–9”) | 0.7 ± 0.4 | (-0.15) ± 0.12 | .21 |  | 1.0 ± 0.4 | (-0.12) ± 0.07 | .11 |
| MCS^a,b^ (“0”, “1”, “2”, “3”) | NA | (-0.19) ± 0.16 | .24 |  | NA | (-0.24) ± 0.16 | .12 |
| Body weight (kg) | 3.9 ± 0.2 | (-0.03) ± 0.01 | .08 |  | 4.0 ± 0.2 | (-0.05) ± 0.02 | **.01** |
| Albumin (g/dL) | 3 ± 0.1 | (-0.01) ± 0.01 | .25 |  | 3 ± 0.1 | (-0.01) ± 0.01 | .49 |
| ALP (U/L) | 28 ± 2 | (-0.3) ± 0.5 | .54 |  | 29 ± 3 | 0.8 ± 0.5 | .15 |
| ALT^b^ (U/L) | 99 ± 30 | (-0.8) ± 1.7 | .65 |  | 51 ± 33 | 1.2 ± 1.6 | .46 |
| Chloride (mEq/L) | 116.8 ± 0.6 | (-0.07) ± 0.24 | .78 |  | 116.8 ± 0.7 | (-0.36) ± 0.26 | .16 |
| Creatinine (mg/dL) | 2.5 ± 0.12 | 0.01 ± 0.03 | .67 |  | 2.5 ± 0.13 | 0.001 ± 0.031 | .97 |
| ln[FGF23] (pg/mL) | 6.05 ± 0.14 | 0.12 ± 0.04 | **.003** |  | 6.02 ± 0.16 | 0.04 ± 0.04 | .35 |
| Glucose (mg/dL) | 126 ± 8 | 3.2 ± 2.3 | .17 |  | 124 ± 9 | 1.5 ± 2.5 | .55 |
| Venous HCO_3_^–a^ (mEq/L) | 21.8 ± 0.5 | (-0.04) ± 0.13 | .72 |  | 22.0 ± 0.5 | 0.12 ± 0.13 | .33 |
| Ionized calcium (mg/dL) | 5.4 ± 0.05 | 0.02 ± 0.03 | .41 |  | 5.31 ± 0.05 | (-0.06) ± 0.03 | .06 |
| PCV (%) | 34 ± 1 | (-0.4) ± 0.3 | .17 |  | 33 ± 1 | (-0.2) ± 0.3 | .4 |
| Venous pH | 7.36 ± 0.01 | (-0.001) ± 0.002 | .73 |  | 7.38 ± 0.01 | 0.001 ± 0.002 | .58 |
| Phosphate (mg/dL) | 3.82 ± 0.11 | 0.06 ± 0.05 | .2 |  | 3.59 ± 0.12 | 0.09 ± 0.05 | .08 |
| Potassium (mEq/L) | 4.04 ± 0.09 | 0.02 ± 0.04 | .65 |  | 3.90 ± 0.10 | 0.03 ± 0.04 | .46 |
| ln[PTH]^b^ (pg/mL) | 2.40 ± 0.19 | (-0.02) ± 0.05 | .72 |  | 2.66 ± 0.21 | 0.03 ± 0.04 | .37 |
| SBP (mmHg) | 133 ± 3 | 0.1 ± 1.3 | .93 |  | 132 ± 3 | 0.9 ± 1.4 | .52 |
| SDMA (μg/dL) | 19 ± 1 | 0.4 ± 0.4 | .3 |  | 19 ± 1 | 0.4 ± 0.4 | .28 |
| Sodium (mEq/L) | 154 ± 1 | 0.2 ± 0.2 | .35 |  | 153 ± 1 | 0.3 ± 0.2 | .24 |
| Total calcium (mg/dL) | 10.38 ± 0.11 | 0.04 ± 0.05 | .39 |  | 10.19 ± 0.12 | (-0.06) ± 0.05 | .27 |
| Total magnesium (mg/dL) | 2.06 ± 0.04 | 0.01 ± 0.04 | .82 |  | 2.06 ± 0.04 | 0.17 ± 0.05 | **<.001** |
| Total protein (g/dL) | 7.9 ± 0.1 | (-0.05) ± 0.04 | .22 |  | 7.8 ± 0.2 | (-0.02) ± 0.04 | .62 |
| Urea (mg/dL) | 46.1 ± 2.3 | 1 ± 0.72 | .17 |  | 47.1 ± 2.6 | (-0.17) ± 0.83 | .84 |

Outcome variables showing significant rate of change in each group (*P* ≤ .05) are highlighted in bold (gradient of regression line significantly different from 0). The unit used for time was month (30.4 days). Results are presented as coefficient (β) ± standard error.

^a^Odds ratio can be obtained by the exponentiation of β and standard error. 95% confidence intervals of the odds ratio can be obtained by the exponentiation of ((β ± 1.96) × standard error).

^b^Only the case number of each individual cat was included as random effect in the model.

Abbreviations: n, number of cats; BCS, body condition score; MCS, muscle condition score; ALP, alkaline phosphatase; ALT, alanine aminotransferase; HCO_3_^–,^ bicarbonate; ln[FGF23], log-transformed fibroblast growth factor-23; OR, odd ratio; PCV, packed cell volume; ln[PTH], log-transformed parathyroid hormone; SBP, systolic blood pressure; SDMA, symmetric dimethylarginine.
