# Supplementary material for: Dietary magnesium supplementation in cats with chronic kidney disease: A prospective double‐blind randomized controlled trial
Source: J Vet Intern Med. 2024 Jul 1;38(4):2180–95. doi: 10.1111/jvim.17134 (PMC11256178; doi:10.1111/jvim.17134)
Supplement: Supplementary file 9 — Table S5. Linear mixed model, generalized estimating equation and generalized linear mixed model analyses examining the change in clinicopathological variables over time in the per‐protocol cats (n = 44) during the study period. Summary of intercepts and the slopes between groups (“control PRD” or “magnesium‐enriched PRD”). [file JVIM-38-2180-s004.docx]

**SUPPLEMENTARY TABLE 5.** Linear mixed model, generalized estimating equation and generalized linear mixed model analyses examining the change in clinicopathological variables over time in the per-protocol cats (n = 44) during the study period. Summary of intercepts and the slopes between groups (“control PRD” or “magnesium-enriched PRD”).

| **Variables** | **Control PRD (n = 33)** | | |  | **Magnesium-enriched PRD (n = 27)** | | |
| --- | --- | --- | --- | --- | --- | --- | --- |
|  | Intercept | Coefficient of time  (β; month) | *P*-value |  | Intercept | Coefficient of time  (β; month) | *P*-value |
| BCS^a, b^ (“1–3”, “4–9”) | 0.99 ± 0.47 | (-0.25) ± 0.14 | .08 |  | 1.21 ± 0.49 | (-0.17) ± 0.13 | .18 |
| MCS^a, b^ (“0”, “1”, “2”, “3”) | NA | (-0.28) ± 0.21 | .17 |  | NA | (-0.24) ± 0.20 | .24 |
| Body weight^a^ (kg) | 3.9 ± 0.2 | (-0.03) ± 0.02 | **.05** |  | 4.1 ± 0.2 | (-0.04) ± 0.02 | **.01** |
| Albumin (g/dL) | 3 ± 0.1 | (-0.01) ± 0.01 | .63 |  | 3 ± 0.1 | (-0.001) ± 0.01 | .97 |
| ALP (U/L) | 27 ± 3 | (-0.2) ± 0.6 | .75 |  | 29 ± 3 | 0.8 ± 0.7 | .26 |
| ALT^b^ (U/L) | 62 ± 9 | (-0.6) ± 2.3 | .8 |  | 55 ± 9 | 2.2 ± 2.4 | .36 |
| Chloride (mEq/L) | 117.3 ± 0.9 | (-0.29) ± 0.31 | .36 |  | 116.7 ± 0.9 | (-0.50) ± 0.34 | .15 |
| Creatinine (mg/dL) | 2.48 ± 0.15 | (-0.002) ± 0.041 | .97 |  | 2.6 ± 0.16 | 0.02 ± 0.05 | .61 |
| ln[FGF23] (pg/mL) | 6.08 ± 0.16 | 0.14 ± 0.05 | **.01** |  | 6.03 ± 0.16 | 0.05 ± 0.06 | .37 |
| Glucose (mg/dL) | 121 ± 7 | 9.4 ± 4.8 | .11 |  | 121 ± 8 | 1 ± 4.9 | .84 |
| Venous HCO_3_^–^ (mEq/L) | 21.6 ± 0.6 | 0.06 ± 0.15 | .69 |  | 21.4 ± 0.6 | 0.51 ± 0.16 | **.004** |
| Ionised calcium (mg/dL) | 5.39 ± 0.05 | 0.08 ± 0.05 | .08 |  | 5.29 ± 0.05 | (-0.09) ± 0.05 | .06 |
| PCV (%) | 34 ± 1 | (-0.4) ± 0.3 | .22 |  | 32 ± 1 | (-0.2) ± 0.4 | .54 |
| Venous pH^b^ | 7.37 ± 0.01 | (-0.001) ± 0.003 | .83 |  | 7.37 ± 0.01 | 0.003 ± 0.003 | .22 |
| Phosphate (mg/dL) | 3.78 ± 0.12 | 0.03 ± 0.07 | .67 |  | 3.62 ± 0.13 | 0.09 ± 0.08 | .25 |
| Potassium (mEq/L) | 4.01 ± 0.11 | 0.004 ± 0.052 | .94 |  | 3.92 ± 0.11 | 0.03 ± 0.06 | .57 |
| ln[PTH] (pg/mL) | 2.35 ± 0.24 | (-0.02) ± 0.06 | .69 |  | 2.87 ± 0.25 | 0.03 ± 0.05 | .54 |
| SBP (mmHg) | 132 ± 3 | (-0.1) ± 1.4 | .94 |  | 134 ± 3 | (-0.4) ± 1.7 | .83 |
| SDMA (μg/dL) | 19 ± 1 | 0.5 ± 0.5 | .38 |  | 20 ± 1 | 0.2 ± 0.6 | .7 |
| Sodium^b^ (mEq/L) | 154 ± 1 | (-0.2) ± 0.3 | .34 |  | 153 ± 1 | 0.3 ± 0.2 | .3 |
| Total calcium (mg/dL) | 10.4 ± 0.12 | 0.1 ± 0.07 | .15 |  | 10.11 ± 0.12 | (-0.06) ± 0.08 | .42 |
| Total magnesium (mg/dL) | 2.03 ± 0.04 | 0.03 ± 0.06 | .69 |  | 2.04 ± 0.05 | 0.25 ± 0.07 | **<.001** |
| Total protein (g/dL) | 7.9 ± 0.2 | 0.01 ± 0.03 | .84 |  | 7.8 ± 0.2 | 0.01 ± 0.03 | .8 |
| Urea (mg/dL) | 44.6 ± 2.6 | 0.82 ± 0.95 | .39 |  | 49.5 ± 2.6 | (-0.29) ± 1.05 | .78 |

Outcome variables showing significant rate of change in each group (*P* ≤ .05) are highlighted in bold (gradient of regression line significantly different from 0). The unit used for time was month (30.4 days). Results are presented as coefficient (β) ± standard error.

^a^Odds ratio can be obtained by the exponentiation of β and standard error. 95% confidence intervals of the odds ratio can be obtained by the exponentiation of ((β ± 1.96) × standard error).

^b^Only the case number of each individual cat was included as random effect in the model.

Abbreviations: BCS, body condition score; MCS, muscle condition score; ALP, alkaline phosphatase; ALT, alanine aminotransferase; ln[FGF23], log-transformed fibroblast growth factor-23; HCO_3_^–,^ bicarbonate; PCV, packed cell volume; ln[PTH], log-transformed parathyroid hormone; SBP, systolic blood pressure; SDMA, symmetric dimethylarginine.
